# Emergency transfemoral valve-in-valve transcatheter aortic valve implantation in patient with chronic type A aortic dissection

**DOI:** 10.1007/s12471-023-01809-9

**Published:** 2023-08-31

**Authors:** Hugo M. Aarts, Astrid C. van Nieuwkerk, Jan Baan, Ronak Delewi, Marcel A. M. Beijk

**Affiliations:** 1 Department of Cardiology, Amsterdam University Medical Centres, Amsterdam, The Netherlands; 2 Department of Utrecht, University Medical Centre Utrecht, Utrecht, The Netherlands

**Fig. 1:**
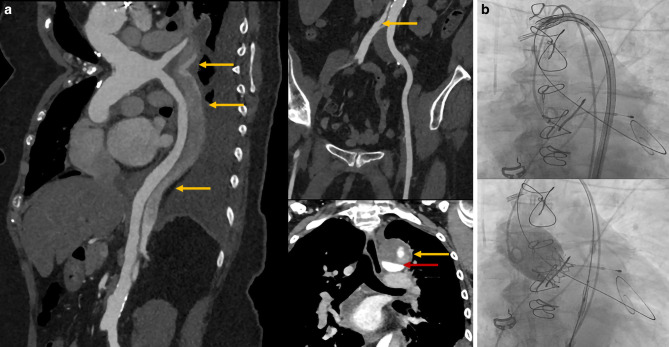
**a** Preprocedural computed tomography scans showing chronic aortic dissection extending into left proximal carotid artery and left subclavian artery, left renal artery and both common iliac arteries (all indicated by *yellow arrows*). Red arrow indicates true lumen of aortic arch. **b** New valve was passed through dissected aortic arch as operators gave traction on guide wire to make sure inner curve was followed. New valve was implanted without any complications

A 71-year-old woman was admitted to our hospital with progressive dyspnoea. She had a history of aortic dissection (DeBakey type I), which was treated with a biological Bentall procedure (Crown 23 mm) and hemiarch replacement in 2016. Echocardiography showed severe valve degeneration and acute deterioration in left ventricular function. Despite optimal medical treatment, she went into cardiogenic shock with multi-organ failure. Redo surgery was considered extremely high risk due to the chronic aortic dissection and ongoing cardiogenic shock (Fig. 1a). Therefore, we performed salvage transfemoral valve-in-valve transcatheter aortic valve implantation (TAVI) under local anaesthesia. An Edwards Sapien 3 Ultra 23-mm valve was successfully implanted without any procedural complications (Fig. 1b). Haemodynamics improved immediately after valve deployment, and after a few days, the patient was discharged home.

This case highlights that transfemoral valve-in-valve TAVI is a treatment option for patients requiring immediate intervention, especially for those who harbour high risk during conventional redo surgery.

